# *Carex pulicaris* abundance is positively associated with soil acidity, rainfall and floristic diversity in the eastern distribution range

**DOI:** 10.1038/s41598-022-06695-6

**Published:** 2022-02-23

**Authors:** Zofia Sotek, Małgorzata Stasińska, Ryszard Malinowski, Renata Gamrat, Małgorzata Gałczyńska, Thea Kull, Sergej Mochnacký, Grzegorz Grzejszczak, Dariusz Paprota, Vladislav Kolarčik

**Affiliations:** 1 Institute of Marine and Environmental Sciences, University of Szczecin, Adama Mickiewicza 16, 70-383 Szczecin, Poland; 2 Department of Environmental Management, Faculty of Environmental Management and Agriculture, West Pomeranian University of Technology in Szczecin, Słowackiego 17, 71-434 Szczecin, Poland; 3 Department of Bioengineering, Faculty of Environmental Management and Agriculture, West Pomeranian University of Technology in Szczecin, Słowackiego 17, 71434 Szczecin, Poland; 4 Institute of Agricultural and Environmental Sciences, Estonian University of Life Sciences, Fr. R. Kreutzwaldi 5, 51006 Tartu, Estonia; 5 Botanical Garden, Pavol Jozef Šafárik University in Košice, Mánesova 23, 04352 Košice, Slovakia; 6 Institute of Biology, University of Szczecin, Wąska 13, 71-415 Szczecin, Poland; 7 Institute of Biology and Ecology, Faculty of Science, Pavol Jozef Šafárik University in Košice, Mánesova 23, 04352 Košice, Slovakia

**Keywords:** Biodiversity, Biogeography, Climate-change ecology, Community ecology, Conservation biology, Wetlands ecology, Ecology, Plant sciences, Plant ecology

## Abstract

*Carex pulicaris* is considered an endangered species, and further losses are forecast under the influence of even moderate climate change. Local studies indicate that temporal declines in *C. pulicaris* abundance are positively correlated to decreases in precipitation and increases in air temperature. Determining ecological properties on larger scales than local ones can help develop effective protection programs for the species. We hypothesize that the local relationships observed between *C. pulicaris* abundance and precipitation, air temperature and soil properties will be confirmed in a spatially-oriented large-scale study performed in situ. Therefore, the present study takes a novel, large-scale integrated approach to (1) precisely characterize the ecological requirements of *C. pulicaris* within its eastern distribution range, and (2) determine the influence of its community type, soil properties and climatic conditions on its abundance. It was found that *C. pulicaris* is not a dominant or codominant species in the studied phytocoenoses in the eastern distribution range. Five natural vegetation groups including *C. pulicaris*, with significantly diverse species compositions, were resolved: well supported Estonian, Polish, Slovak and Radecz groups, and a weakly-supported Ambiguous group. The abundance of *C. pulicaris* was found to be positively correlated with the composition of the geographically-diversified plant communities and atmospheric precipitation, and to be also negatively associated with latitude and soil pH. Although the species is adapted to a relatively wide range of soil types, such adaptation requires appropriate substrate moisture level and light conditions. The species prefers moist organic and mineral soils and grows on both acid and neutral medium, characterized by a narrow C:N ratio, with various amounts of digestible total P, Mg and N, and low levels of digestible K. Climate change, manifested by reduced rainfall, may be one of the most important predictors negatively affecting the occurrence of *C. pulicaris*.

## Introduction

All conservation initiatives are subject to the influence of climate change, which will undoubtedly have a dramatic impact on many ecosystems^1–4^. Such changes will be reflected in species distribution and phenology, as well as in interspecies interactions, among others, and they are expected to become the main threat to biodiversity. Of these species, the most vulnerable will be the rare and endangered ones, as they are usually characterized by a narrower niche width^5^. Therefore, one of the core goals of conservation biology is to understand the distribution, abundance and ecological requirements of rare species^6,7^. Such studies are urgently needed for species whose natural habitat is fragmented and vulnerable under anthropogenic pressure. Some regional differences related to climate, geology and the history of land use may occur within the species range^8,9^. It is also important to note that conservation efforts should focus not only on populations from the centers of their distribution, but even more on those located at the edges, where the risk of extinction is highest^10^.

Global climate change has increased the risk of summer droughts, which makes fen habitats one of the most sensitive habitats in Europe. In many cases, this damage is exacerbated by anthropogenic land transformations of varying intensity, such as extensive landscape degradation and drainage. Unfortunately, habitat restoration programmes may fail to recover the biodiversity of degraded peatlands^11^. As a result, many fen species are believed to be at risk of extinction, and species conservation has become a priority in wetland ecosystems.

Wetland ecosystems, and peatlands in particular, are a refuge for many rare plant species associated with specific habitat conditions and which significantly enrich the biodiversity of a given region. Many of these are stenotopic organisms, with a very narrow ecological niche. As a result of the transformation and disappearance of peatland habitats, the population of these plants decreases, which, in turn, may lead to the disappearance of their localities and their withdrawal from previously-occupied areas. Such losses may cause disjunctions within their species range and, in drastic cases, even a shrinkage of the range itself^12^.

*Carex pulicaris* (flea sedge) is a typical fen species, occurring in western and northern Europe, ranging from Spain and Ireland in the west to Scandinavia in the north, with its eastern distribution limits in Baltic countries (Estonia, Lithuania, Latvia and Poland), Belarus and central Europe (Slovakia); the species does not reach the Mediterranean region^13^. The occurrence of *C. pulicaris* has a fragmented, island-like character at the eastern edge of its distribution range, with a disjunction in Poland east of the Bay of Puck between Atlantic and Eastern Baltic sites. Several studies have already documented a loss of *C. pulicaris* localities in recent decades, e.g. in southern Germany, in Slovakia, northwest Poland and Estonia^14–17^. Indeed, the species is classified in various threat categories in many European countries, being considered endangered in Central Europe^18–20^ or vulnerable in Fennoscandia^21,22^. In Estonia, according to the last Red Data Book assessment, the species is near threatened^23^.

Populations of *C. pulicaris* are mainly associated with wet and periodically wet habitats, and occasionally strongly transformed ones. They occur in low and transitional bogs, laggs of raised bogs, peaty meadows and wet forest habitats, and less often on impermeable loam and clay slopes^14,15,24^. The species is found on both acidic and neutral soils, preferring peat soils. It is less common on soil-glial soils^7,15,16^.

*Carex pulicaris* does not usually contribute much to the construction of plant communities. It is considered a species typical for the Caricetalia davallianae order and the Caricion davallianae alliance^25^. It is a frequent element of plant communities classified into the new, recently-distinguished Sphagno warnstorfiani—Tomenthypnion alliance^26,27^, belonging to the Caricetalia davallianae order. It is also a part of the Caricion nigrae and Caricion lasiocarpae alliance of the Scheuchzerio-Caricetea nigrae class, and encroaches into the Calthion and Molinion alliance of the Molinio-Arrhenatheretea class^7,15,25,28,29^.

*Carex pulicaris* is a highly vulnerable species. Distribution models suggest that it will be lost from many regions along its eastern distributional limits in response to moderate climate change^30^. Further studies also suggest that assisted migration may be a beneficial management strategy for the species, with reintroduction being another potential management strategy^31^. As the species is a weak competitor, low nutrient availability and low competition for light may favour its long-term survival. A case study performed at the regional scale found that negative changes in hydrological regimes occurring since 1950, influenced mainly by lower summer precipitation and higher mean annual temperature, both driven by climate change, are significantly associated with predicted extinctions of *C. pulicaris* populations occurring at lower altitude limits^14^. However, all these results were obtained from modelling based on climate-only data or regional studies. There is a lack of detailed studies of habitat conditions across a larger area of occurrence; such research is extremely important, especially at the edges of the distribution range, where the species is less likely to find favorable conditions for development, and its sites are more vulnerable to disappearance.

Hence, there is a pressing need to identify the factors determining the abundance of *C. pulicaris* with more comprehensive studies of the habitat parameters and floristic diversity of plant communities over a larger area, including the climate conditions. Such data can be used to identify appropriate protective measures to prevent the emergence of disjunctions and the projected shrinkage of the eastern range as a result of projected climate change. We expect that in the eastern range of *C. pulicaris*, its abundance is significantly influenced by temperature and rainfall^14^, vegetation type and substrate properties (soil type, organic matter content, nutrient abundance and soil acidity). We also hypothesise that both the abundance of *C. pulicaris* and the diversity of plant communities in which it occurs may be related to the region of its occurrence.

The present study addresses the following questions:

1. Do the plant communities in which C. pulicaris occurs vary regionaly, and is species abundance related to community diversity?

2. How can climatic factors affect the communities of *C. pulicaris* and its abundance at the eastern end of its range, with regard to different latitudes and climatic conditions?

3. What are the properties of the soil in the eastern range of *C. pulicaris*, and do they affect its abundance?

## Materials and methods

### Phytosociological data

**Figure 1:**
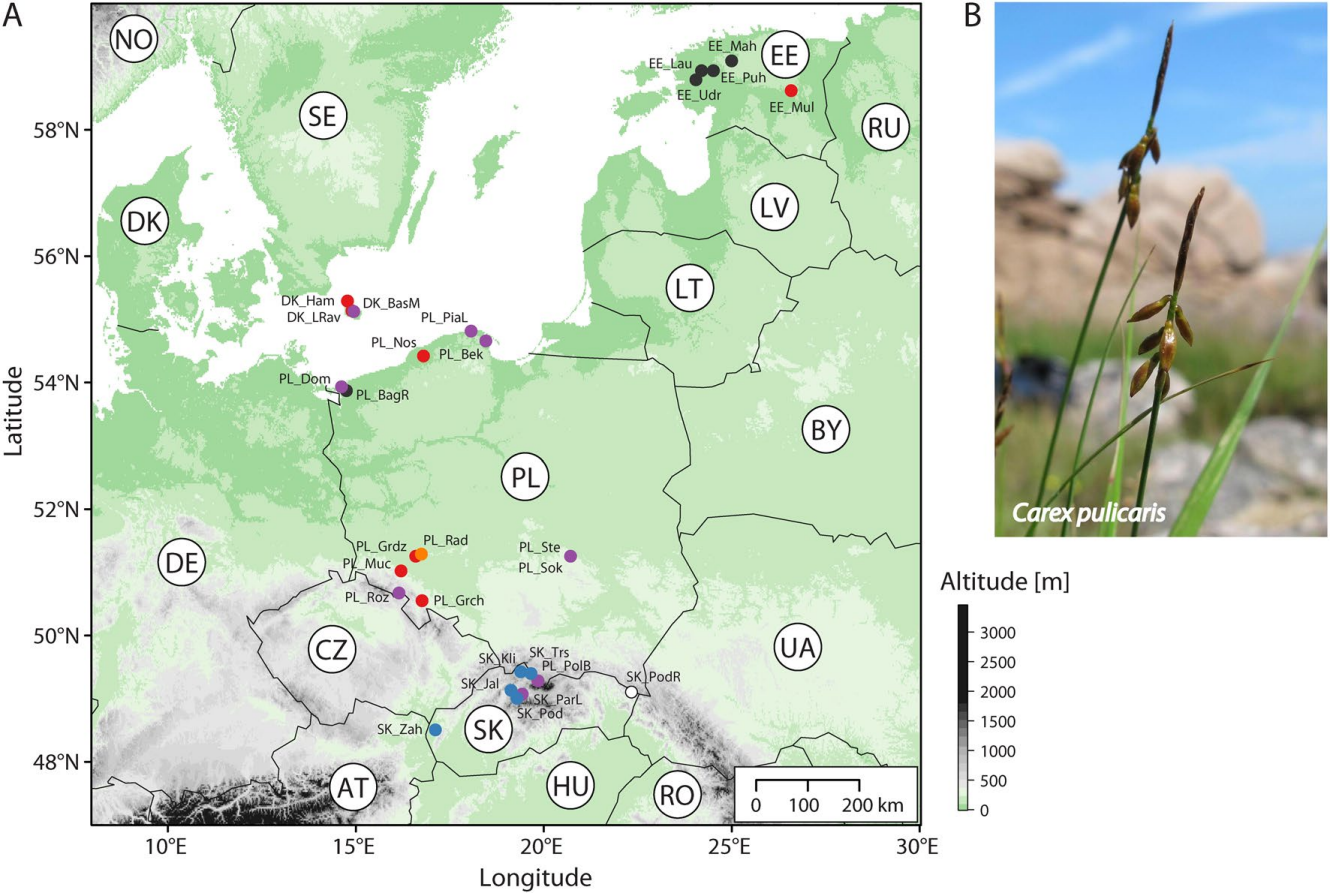
Survey of investigated localities with the occurrence of *Carex pulicaris*. (**A**) Distribution map of sampled sites, colouring according to plant communities as inferred in the present study; (**B**)* Carex pulicaris* (photo: Grzegorz Grzejszczak). Countries are indicated using two-letter ISO 3166-1 alpha-2 codes. Locality abbreviations follow Table 1. Colouring of symbols: black—Estonian group, blue—Slovakian group, orange—Radecz group, purple—Ambiguous group, red—Polish group, white—locality in Slovakia with the occurence of *C. pulicaris*, but lacks species composition data.

Field tests of the conditions favouring the occurrence of *C. pulicaris* were performed at 17 different locations: three in Denmark (Bornholm), six in Poland and six in Estonia, and two in Slovakia (Fig. 1, Table 1).

**Table 1:** Localities and selected climatic factors.

Country	Locality	Latitude (N)	Longitude (E)	Mean annual precipitation [mm]	Min. day temp.^a^ [°C]	Mean day temp.^b^ [°C]	Mean day temp. Dec/Jan^c^ [°C]
Estonia	Mahtra (EE_Mah)^d^	59° 05.312ʹ	25° 00.129ʹ	471	− 19	9	− 8
	Pühatu (EE_Puh)^d^	58° 56.371ʹ	24° 30.548ʹ	453	− 19	9	− 8
	Üdruma (EE_Udr)^d^	58° 47.292ʹ	24° 03.069ʹ	408	− 18	9	− 8
	Laukna (EE_Lau1, EE_Lau2)^d^	58° 55.957ʹ	24° 11.379ʹ	423	− 18	10	− 6
	Mullavere (EE_Mul)^d^	58° 37.172ʹ	26° 34.419ʹ	402.5	− 18	9	− 10
Poland	Domysłów (PL_Dom)^d^	53° 55.976ʹ	14° 37.321ʹ	351.5	− 4	13	− 2
	Beka (PL_Bek)^d^	54° 39.379ʹ	18° 27.699ʹ	329	− 9	11	− 1
	Nosalin (PL_Nos2)^d^	54° 25.198ʹ	16° 47.798ʹ	420	− 7	12	− 2
	Rozwarowskie Marsh (PL_BagR)^d^	53° 52.376ʹ	14° 45.075ʹ	364.5	− 5	12	− 1
	Piaśnickie Łąki (PL_PiaL)^d^	54° 49.388ʹ	18° 3.844ʹ	352.5	− 8	12	− 1
	Polana Biały Potok (PL_PolB)^d^	49° 17.031ʹ	19° 50.792ʹ	915	− 12	9	− 7
	Różana (PL_Roz)	50° 40.483ʹ	16° 09.150ʹ	603	− 11	12	− 8
	Grochowa (PL_Grch)	50° 33.152ʹ	16° 45.371ʹ	538	− 10	13	− 4
	Muchów (PL_Muc)	51° 01.166ʹ	16° 01.200ʹ	545.5	− 12	12	− 2
	Grodzanów (PL_Grdz)	51° 15.200ʹ	16° 35.617ʹ	486.5	− 8	14	− 1
	Stefanków (PL_Ste)	51° 15.447ʹ	20° 42.646ʹ	507.5	− 12	13	− 4
	Sokołów (PL_Sok)	51° 30.742ʹ	18° 44.591ʹ	507.5	− 12	13	− 4
	Radecz (PL_Rad)	51° 17.419ʹ	16° 44.435ʹ	486	− 9	13	− 1
Denmark (Bornholm)	Sandviq (Hammerk) (DK_Ham)^d^	55° 17.678ʹ	14° 46.700ʹ	310	− 2	9	2
	Ravnekaer Lake (DK_LRav)^d^	55° 08.329ʹ	14° 54.127ʹ	325	− 6	11	− 5
	Bastemosse (DK_BasM)^d^	55° 07.406ʹ	14° 56.685ʹ	325	− 7	11	− 3
Slovakia	Ružomberok-Podsucha 1 (SK_Pod1)^d^	49° 00.163ʹ	19° 17.290ʹ	727	− 10	11	− 8
	Partizánska Ľupča (SK_ParL)	49° 04.147ʹ	19° 25.504ʹ	687.5	− 15	14	− 10
	Jalovec (SK_Jal)	49° 08.000ʹ	19° 07.700ʹ	721.5	− 8	11	− 6
	Záhorska Nížina (SK_Zah)	48° 30.667ʹ	17° 06.566ʹ	566	− 8	14	− 3
	Klinské rašelinisko (SK_Kli)	49° 25.766ʹ	19° 23.734ʹ	785.5	− 12	8	− 7
	Trstená (SK_Trs)	49° 23.917ʹ	19° 00.040ʹ	749.5	− 14	13	− 9
	NP Poloniny NPR Pod Ruským (SK_PodR)	49° 06.229ʹ	22° 19.949ʹ	682.5	− 9	12	− 5

^a^Minimum value of the 8-day MODIS day-time LST time series data.

^b^Mean value the 8-day MODIS day-time LST time series data.

^c^Mean value of the 8-day MODIS day-time LST time series data for Dec/Jan.

^d^Localities where the authors made phytosociological relevés and took soil samples.

Phytosociological relevés were performed in patches of *C. pulicaris* communities, using the classic Braun-Blanquet method based on a seven-degree quantitative scale. Statistical analyses were performed on newly-gathered phytosociological data (17 relevés), other phytosociological relevés of Poland (13 relevés), which are recorded in the Polish Vegetation Database^32^, as well as previously published data from Slovakia (13 relevés)^29^. In total, our analyses were based on 43 phytosociological relevés recorded from 27 localities (1–3 per locality). The syntaxonomic approach to communities was adopted after Matuszkiewicz (2006)^25^.

The habitat conditions were assessed based on Ellenberg indicator values (EIVs)^33^: ecological (L—light value, T—temperature value, K—continental value, F—humidity value, R—acidification value, N—nitrogen value, S—salinity value), sociological (Gr—group of vegetation classes, K.1—class, o—order, v—alliance, u—suballiance) frequency and risk (m—frequency of appearance, D—domination, A—the trend of change, G—threats). In the case of 17 of the examined locations, the soil data were also analysed.

### Climatic data

Climatic conditions were evaluated for all the analysed locations based on the rasters collected in the https://worldgrids.org library, developed for the needs of the “Soilgrids” project, organized by the International Soil Reference and Information Centre (ISRIC, https://www.isric.org/explore/soilgrids). The data are currently available in the archive at https://web.archive.org/web/20170619054443/http://www.worldgrids.org/doku.php/start.

The analyses included latitude, longitude, daily minimal temperatures, daily mean temperatures, daily mean temperatures in December/January, and annual mean monthly precipitation (Table 1). The raster resolution for temperature data was 1 km, and 5.6 km for rainfall data.

### Chemical composition of soil samples

Composite samples were collected from the surface layer of the rhizosphere (0–20 cm) for each of the natural sites^34^ (Table 1). In the soil material, the following analyses were performed: loss on ignition (organic matter) by burning soil samples in a muffle furnace at the 550º C; total C, N and S content by elementary analysis (Costech Elementary Analyzer ECS 4010, Italy); pH in H_2_O and pH in 1 mol dm^−3^ KCl was determined potentiometrically; salinity by conductometry.

In addition, the content of Mg, K, Ca, Na, Cd, Co, Cu, Ni, Pb, Mn, Fe and Zn soluble in 0.5 mol dm^−3^ HCl (the so-called available forms)^35,36^ and soluble in concentrated HNO_3_ and HClO_4_, at the ratio 1:1 (the so-called total forms) was determined using an ICE series 3000 spectrometer with flame atomization (FAAS)^37^. The content of Na, Ca and K was determined by flame atomic emission spectrometry, while that of the other elements was determined using flame atomic absorption spectrometry. The limits of detection were (mg kg^−1^): Ca—0.004; Mg—0.002; K—0.001; Na—0.004; Fe—0.004; Cd—0.003; Co—0.010; Cu—0.005; Ni—0.008; Pb—0.013; Zn—0.003 and Mn—0.002. The content of available and total P was determined with spectrophotometric molybdenum blue method (690 nm wave length)^37^ using a Marcel MEDIA™ spectrophotometer.

The accuracy and precision of the analytical methods and procedures used were confirmed using certified reference material: CRM036-050 Loamy Sand 4 (CRM 036-050 produced by Resource Technology Corporation, USA and UK). The effectiveness of the process was validated with 90–95% efficiency. The results shown are the mean values of three measurements, with working standards made from Merck standards at a concentration of 1000 mg dm^−3^.

The soil type classification follows the international and regional standards^38–40^.

### Statistical analyses

The abundance of *Carex pulicaris* was compared with the phytosociological composition of sites and three sets of environmental variables: mean Ellenberg indicator values (EIVs), climate variables (CLIM) and soil variables (SOIL).

The species composition and soil types of the sites were compared by multivariate analyses using the *vegan* package in R environment: non-metric multidimensional scaling (NMDS) of the species composition data matrix (43 sites × 310 species), and principal component analysis (PCA) of the soil data matrix (17 sites × 34 soil variables)^41,42^. The raw species composition data matrix and Bray dissimilarity index were subjected to NMDS, and raw data were centred and scaled prior to PCA. Cluster analysis (normalised data, Ward agglomerative method) was performed to delineate natural phytosociological groups; the grouping pattern was used throughout results. The relationship of *C. pulicaris* to site structure and its relationship with possible explanatory variables were assessed by fitting *C. pulicaris* abundance data over NMDS or PCA ordinations using the envfit function of the *vegan* package^41,42^.

In addition, species composition on the sites was compared with environmental data. The collected data were utilised to construct six data matrices; three types of environmental data matrices: (1) mean Ellenberg indicator values (EIVs), (2) climate variables, (3) soil variables, and three corresponding species-composition data matrices. The species composition data in each locality were averaged per locality to obtain a reduced dataset corresponding to database-mined climate data (reduction from 43 sites to 27 localities); this dataset was independent of the species composition in each location.

Canonical correspondence analysis (CCA) was performed in *vegan*^41,42^ to formally test for the ecological interpretation (EIVs and CLIM) of the sites and the species similarity assemblages. ANOVA was applied for statistical testing, and the global CCA model was statistically significant. The parsimonious CCA model was selected by a permutation test using forward model selection.

SOIL data were not subjected to CCA, since the number of sites (n = 16, with available plant community and soil variable data) was much lower than the number of soil variables (n = 34). Instead, it was tested whether soil data may explain species composition data; briefly, the envfit function was used (in *vegan*^41,42^) to superimpose SOIL variables over NMDS ordination of species composition data (n = 16 sites in total).

To characterise each of the localities, plant diversity indices, i.e. the number of species recorded per relevé, Shannon’s H diversity index, and Pielou`s Evenness were also calculated in the *vegan* package of R or in Past 3.10^41–43^. One-way analysis of numerical variance (ANOVA) and Tukey's HSD pairwise multiple comparison were used to test for differences between means of particular parameters of different vegetation groups. Prior to analysis, assumptions of ANOVA, data normality and homogeneity of variance, were tested applying the Shapiro–Wilk test and Levene's test, respectively. Slight deviations from data normality were tolerated. The level of significance was set at p < 0.05 in all analyses. Statistical analysis of the obtained results was performed using R or in Past 3.10^42,43^.

Regarding the tested chemical properties of the soil*,* the statistical significance of the differences between means was determined by testing the normality of distribution in each group and homogeneity of variance in all groups, followed by ANOVA with Tukey's post hoc test. The significance was set at p < 0.05. These analyses were performed using Statistica 12.5 PL software (StatSoft Inc*.*, Tulsa, OK, USA).

## Results

### Vegetation types with the occurrence of *C. pulicaris*

**Figure 2:**
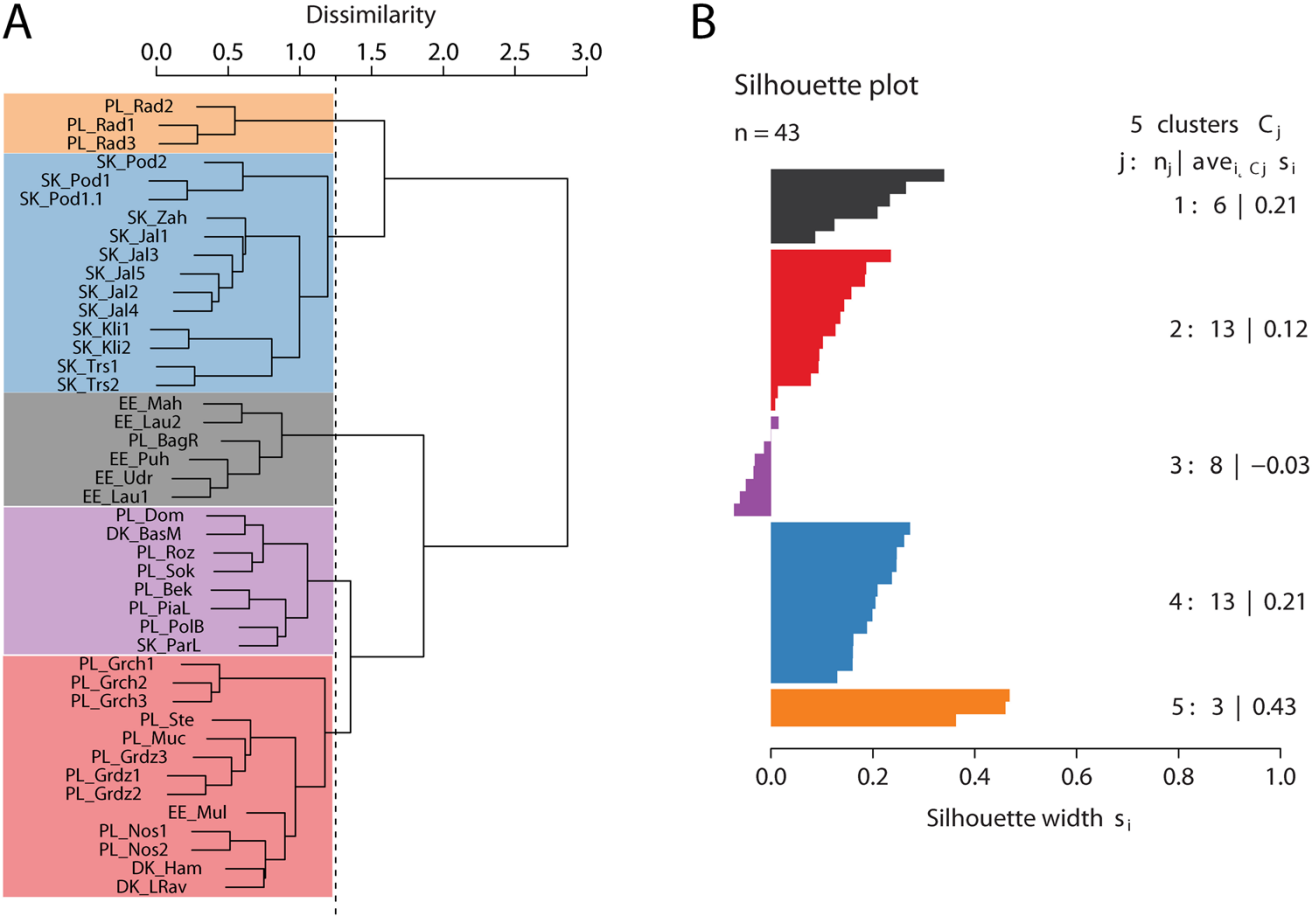
Analysis of vegetation similarity between relevés. (**A**) Cluster analysis by Ward agglomeration; (**B**) The corresponding Silhouette plot allows cluster patterning and significance to be evaluated. Locality abbreviations follow Table 1. Colouring of clusters: black—Estonian group, blue—Slovakian group, orange—Radecz group, purple—Ambiguous group, red—Polish group.

In total, 310 moss and vascular plant species were recorded (59 mosses, 251 vascular plants). *Carex pulicaris* locations were found to demonstrate a range of species compositions. Our cluster analysis of the similarity in species composition between sites (Fig. 2) revealed five natural vegetation groups, as presented throughout the manuscript. These groups roughly correspond to the region of origin: Estonian sites (Estonian group), Polish sites including Danish sites (Polish group), Slovakian sites (Slovak group), relevés from Radecz in Poland, which appear as a separate group (Radecz group) and a final group composed of sites of different regions (Ambiguous group); not surprisingly, the final group has low statistical support (Fig. 2).

Most of the recorded species are very rare in the dataset, with 69.03% being present in fewer than five plots, i.e. approximately 10% of all of investigated plots. In contrast, only ten species are present in more than half of all plots with *C. pulicaris*. These are *Potentilla erecta* (83.37% of all analysed plots), *Carex panicea* (83.72%), *Briza media* (79.07%), *Succisa pratensis* (65.12%), *Molinia caerulea* (62.79%), *Galium uliginosum* (55.81%), *Cirsium palustre* (53.49%), *Calliergonella cuspidata* (51.16%), and *Holcus lanatus* (51.16%).

Other species were found to accompany *C. pulicaris* in particular vegetation types. *Carex davalliana* dominates in the Estonian and Slovak groups (83.33% and 100% of sites, respectively), *Anthoxanthum odoratum* is common in the Polish and Ambiguous groups (92.31% and 75%, respectively), and *Lotus uliginosus* is also common in the Polish group (76.92%). The Slovak group is floristically very rich (results presented below), with *Campylium stellatum* (92.31%), *Fissidens adianthoides* (92.31%), *Bryum pseudotriquetrum* (76.92%), *Eriophorum angustifolium* (76.92%), *Carex echinata* (69.23%), *Carex flava* (69.23%), *Parnassia palustris* (69.23%) and *Plagiomnium elatum* (69.23%) commonly found together with *C. pulicaris*. Many species are commonly observed with *C. pulicaris* in the Radecz group, but these data were obtained from only three related relevés.

**Figure 3:**
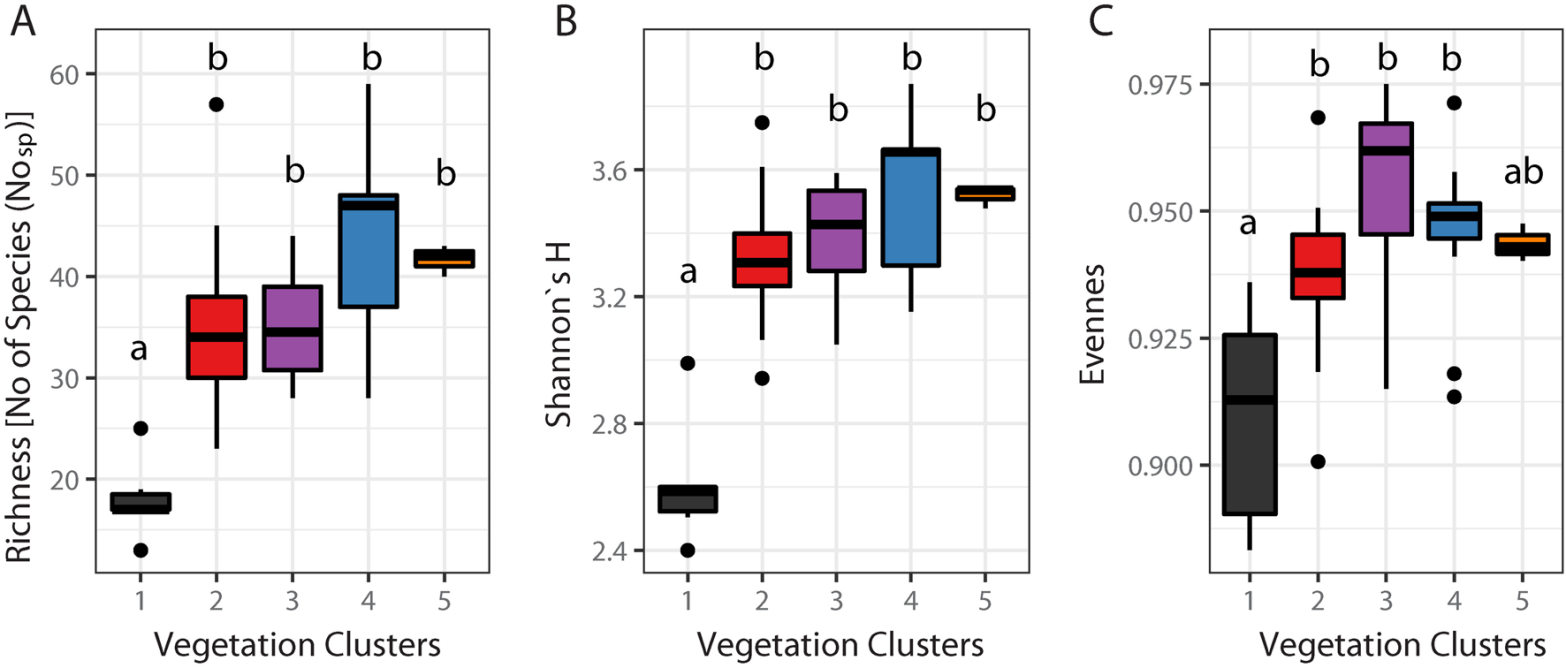
Boxplots of variation of diversity indices in vegetation aggregation. (**A**) Richness, number of species recorded per relevé; (**B**) Shannon`s H diversity index; (**C**) Pielou`s evenness. Statistically homogeneous groups are indicated by lower case letters. Colouring follows clustering pattern: black—Estonian group, blue—Slovakian group, orange—Radecz group, purple—Ambiguous group, red—Polish group.

Statistically significant differences in diversity between natural vegetation groups were found for species richness, Shannon`s H diversity index, as well as for evenness index. The Estonian group differs from all other groups in species richness and Shannon`s H diversity index (Fig. 3). Our data showed that the phytocoenoses with *C. pulicaris* are moderately rich, with the number of species per relevé varying between 13 and 59. Comparatively high species richness was also observed in the Polish (35.38 ± 8.62), Ambiguous (35.00 ± 5.55), Slovak (42.92 ± 9.31) and Radecz groups (41.67 ± 1.53), but lower in the Estonian group (18.00 ± 3.95) (Fig. 3). Shannon`s H diversity index, reflecting species richness, ranged from 2.40 to 3.87 between groups, being high in the Polish (3.32 ± 0.21), Ambiguous (3.39 ± 0.20), Slovak (3.53 ± 0.23) and Radecz groups (3.52 ± 0.03), but lower in the Estonian group (2.61 ± 0.20). The evenness index varies from 0.88 to 0.98, and is always higher than 0.90 within a particular group (Fig. 3), suggesting that species composition has quite an even distribution between sites.

Mosses are prevalent in the Radecz (11–15 species per site, mean 12.3) and Slovak groups (7–18 species per site, mean 11.3); however, they are rare or even absent in the rest, where number of moss species per site ranged from 0 to 8.

### Environmental data assessment of sites with *C. pulicaris* with regard to their vegetation composition and the abundance of *C. pulicaris*

**Figure 4:**
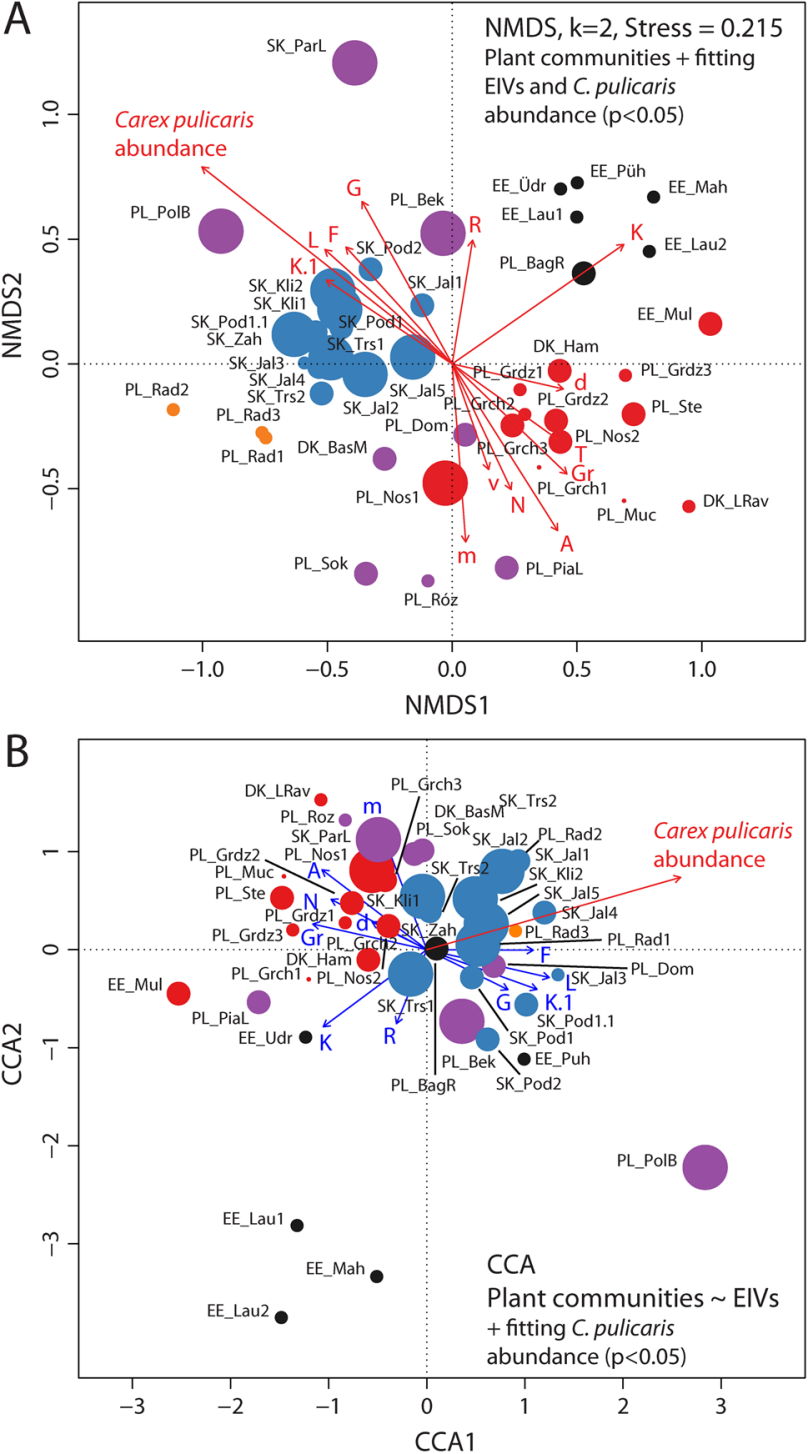
Ordination biplot of non-metric multidimensional scaling (NMDS) and canonical correspondence analysis (CCA). (**A**) NMDS of species composition data showing grouping of localities with maximal variance explained along NMDS1 and NMDS2. Ellenberg indicator values (EIVs, p < 0.05) fitted to ordination biplot; (**B**) Canonical correspondence analysis (CCA) performed on matrices of species composition data and mean EIVs. Only significant variables (p < 0.05) explaining constrained variation, identified based on forward selection, were used in parsimonic CCA. Locality abbreviations follow Table 1. Colouring corresponds to clustering pattern, symbol size reflects *Carex pulicaris* abundance. *Carex pulicaris* abundance vector is fitted to NMDS and CCA models (p < 0.05). Colouring of symbols: black—Estonian group, blue—Slovakian group, orange—Radecz group, purple—Ambiguous group, red—Polish group.

The NMDS grouping of the whole dataset corresponds to the cluster analysis. Four groups, viz*.* the Estonian, Slovak, Polish and Radecz groups, are compact and well resolved on the NMDS ordination biplot (Fig. 4A); however, the members of the Ambiguous group, weakly supported in cluster analysis, are scattered across the NMDS ordination biplot. The abundance of *Carex pulicaris* is significantly correlated with the NMDS ordination of plant communities. CCA performed on mean EIV scores with a forward selection procedure revealed 11 statistically-significant variables (p < 0.05) out of a total of 16 (Fig. 4B). Reducing the set of EIV variables in the CCA explains 37.86% (constrained) of the total variance.

Most of the relevés are located in the upper half of the CCA plot (Fig. 4B). Two groups dominate: the Polish group on the left side of the CCA plot and the Slovak group on the right. The relevés from Radecz are nested within the Slovak group, those of the Ambiguous group are scattered across ordination, while those of the Estonian group are found on the left and lower part of the plot. The EIV indicators suggest that the left–right separation of relevés derives mostly from the presence of species with higher values for Light, sociological class and Humidity (all positively associated with CCA1), as well as Continentality, sociological group of vegetation classes, trend of change and Nitrogen (negatively associated with CCA1). The relevés are separated across the second CCA2 axis by soil reaction and frequency of appearance. The abundance of *C. pulicaris* is also significantly correlated with the CCA-constrained ordination of plant communities. In general, NMDS and CCA correspond to each other and the abundance of *C. pulicaris* is significantly associated with vegetation type, whose differentiation seems to be driven by factors acting on large geographic scales.

**Figure 5:**
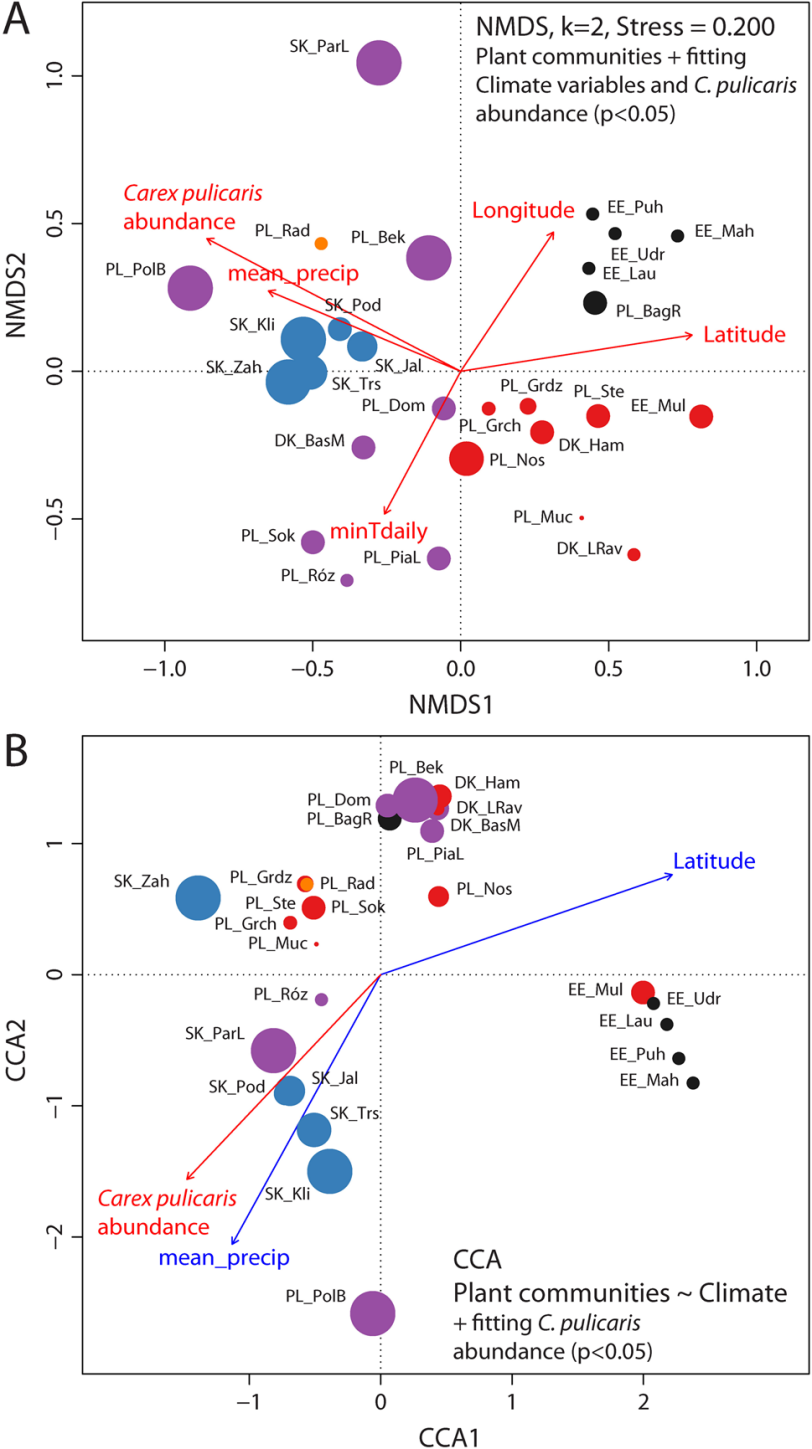
Ordination biplot of non-metric multidimensional scaling (NMDS) and canonical correspondence analysis (CCA). (**A**) NMDS of species composition data showing grouping of localities with maximal variance explained along NMDS1 and NMDS2. Climate variables significant at p < 0.05 fitted to ordination biplot; (**B**) canonical correspondence analysis (CCA) performed on matrices of species composition data and Climate variables. Only significant variables (p < 0.05) explaining constrained variation, identified based on forward selection, were used in parsimonic CCA. Locality abbreviations follow Table 1. Colouring corresponds to clustering pattern, symbol size reflects *Carex pulicaris* abundance. *Carex pulicaris* abundance vector is fitted to NMDS and CCA models (p < 0.05). Colouring of symbols: black—Estonian group, blue—Slovakian group, orange—Radecz group, purple—Ambiguous group, red—Polish group.

The NMDS analyses of the species composition data of the reduced dataset (27 localities, Fig. 5A) found the overall groupings identified in the NMDS results of the whole dataset to be preserved. Latitude, Longitude, Mean precipitation and Minimal daily temperature, as well as *C. pulicaris* abundance, were found to significantly correlate with NMDS ordination.

The CCA analyses performed on the independently-gathered database of climate data described above (Fig. 5B) found that while the reduced CCA model was statistically significant (p = 0.001), only the first two axes were statistically significant (p < 0.05). The forward selection procedure suggests that only Latitude and Mean precipitation are statistically significant variables (p < 0.01). However, this CCA model explains only 11.24% (constrained) of the total variance. The resulting general grouping pattern of vegetation relevés appears slightly changed, with the Estonian group and Polish relevés being found in the right and upper parts of the plot, and the Slovakian group in the lower left part (Fig. 5B). The abundance of *C. pulicaris* was found to fit significantly to the groupings observed in the reduced CCA model; it also correlated negatively with Latitude, which is reflected on CCA ordination (Fig. 5B).

### Soil properties at sites with *C. pulicaris*

The studies on soils associated with *C. pulicaris* in Estonia, Poland, Denmark and Slovakia indicate that the species prefers moist habitats: peat bogs with various degrees of decay (Murshic Histosols) and humus mineral soils (Umbric Gleysols).

In Poland and Estonia, some sites were located in fens with a weakly moorsh forming process. At the surface level (rhizosphere), they were characterized by low ash content (< 20%) and acidic or slightly acidic pH. In Slovakia, *C. pulicaris* also grew in fen, but strongly decayed bogs with high ash content (> 40%) and slightly acidic or neutral pH. The rhizospheres of the bogs differed significantly with regard to total nitrogen content (Table 2). The soils from Estonia contained on average about 80% more total nitrogen than those in Poland and Slovakia. No significant differences were found in mean total sulfur content, C/N ratio or salinity. The soils on which *C. pulicaris* occurred were characterized by a varying content of available phosphorus (from small to medium amounts) and magnesium (from small to very large amounts) and a very low content of available potassium. The Estonian, Polish and Slovak bogs did not differ significantly in their available K or Na content. The peatlands from Poland contained significantly more available P than those from Slovakia, which were richer in available Ca and Mg (Table 2).

**Table 2:** Basic soil properties and macronutrient contents.

The localizations of *Carex pulicaris*		Organic matter		Total			C/N	pH			Salinity	Available macroelements (soluble in 0.5 mol dm^−3^ HCl)				
				C	N	S						P	Na	K	Ca	Mg
		%						H_2_O		KCl	µS cm^−1^	mg kg^−1^				
Estonia	*Organic soils (Murshic Histosols)—Eo*															
	*Laukna 1*	80.28		40.37	2.96	0.34	13.77	6.49		6.02	353.33	191.37	98.63	152.33	3930	1170.25
	*Mullavere*	72.30		36.66	2.63	0.36	14.17	6.91		6.53	140.00	204.36	94.82	158.1	3954	1872
	*Pȕhatu*	87.76		42.87	2.69	0.28	16.01	6.43		5.70	176.67	183.6	69.93	71.4	928.63	126.17
	$\bar{x}$	80.11c		39.97c	2.76d	0.33a	14.65a	6.61bc		6.08bcd	223.33a	193.11ab	87.79a	127.28ab	2937.54a	1056.14abc
	*Mineral soils (Umbric Gleysols)—Em*															
	*Laukna 2*	12.23		5.48	0.34	0.04	15.95	6.83		6.24	135.00	217.35	53.25	145.05	5293.25	889.75
	*Mahtra*	18.29		8.73	0.73	0.16	11.84	7.00		6.29	380.00	247.8	41.05	53.85	1294.07	722.67
	*Ȕdruma*	17.61		8.96	0.69	0.10	12.38	7.01		6.21	130.00	150.37	59.77	111.07	1616.08	1093.13
	$\bar{x}$	16.04a		7.72a	0.59abc	0.10a	13.39a	6.95b		6.25cd	215.00a	205.17ab	51.36a	103.32ab	2734.47a	901.85ab
Denmark (Bornholm)	*Mineral soils (Umbric Gleysols)—Dm*															
	*Sandviq*	5.93		2.73	0.23	0.05	11.87	5.47		4.70	114.00	165.2	35.3	14.72	237.1	55.76
	*Ravnekaer Lake*	14.69		7.32	0.45	0.13	16.27	5.83		5.27	396.00	127.8	56.3	34.01	508.5	152.4
	*Bastemosse*	11.23		5.92	0.38	0.10	15.58	5.94		5.40	354.00	231.1	42.7	16.72	3002	102.3
	$\bar{x}$	10.62a		5.32a	0.35a	0.09a	14.57a	5.75a		5.12a	288.00a	174.70ab	44.77a	21.82a	1249.20a	103.49a
Poland	*Organic soils (Murshic Histosols)—Po*															
	*Domysłów*	84.38		46.42	1.22	0.22	38.05	5.91		5.03	150	284.6	156.3	87.8	768.2	249
	*Rozwarowskie Marsh*	98.18		48.52	0.8	0.07	60.65	5.78		5.16	423	310.3	77.9	321.9	1109	108.1
	*Beka*	72.17		35.32	2.55	0.6	13.85	6.3		5.83	678	417.2	916.2	260.2	6265	401
	$\bar{x}$	84.91c		43.42c	1.52ab	0.30a	38a	5.99a		5.34ab	417a	337.37b	383.47a	223.30b	2714.07a	252.70a
	*Mineral soils (Umbric Gleysols)—Pm*															
	*Nosalin*	12.66		6.00	0.38	0.02	15.79	6.04		5.65	116	631	175.2	53.3	2085.3	135.8
	*Piaśnickie Łąki*	7.65		5.07	0.37	0.09	13.70	5.68		5.14	104	567.3	217.3	30.7	1422.3	165.2
	*Polana Białego Potoku*	12.27		5.38	0.43	0.14	12.51	5.47		4.7	1114	381.7	703.3	237.3	764.5	501.6
	$\bar{x}$	10.86a		5.48a	0.39bc	0.08a	14.00a	5.73a		5.16a	444.67a	526.67c	365.27a	107.10ab	1424.03a	267.53a
Slovakia	*Organic soils (Murshic Histosols)—So*															
	*NP Poloniny NPR 1*	50.36		20.24	1.22	0.26	19.17	6.04		5.83	824.00	124.3	80.6	108.18	10,139.1	2091
	*NP Poloniny NPR 2*	51.86		22.82	1.52	0.32	18.31	6.07		5.87	820.50	114.4	74.75	82.38	9455.25	1787
	*NP Poloniny NPR 3*	52.21		23.71	1.62	0.33	14.78	6.13		5.86	867.00	103.4	78.45	134.23	9187	1825.5
	$\bar{x}$	51.48b		22.25b	1.45abc	0.30a	17.42a	6.08ac		5.85abc	837.17a	114.03a	77.93a	108.26ab	9593.78a	1901.17bc
	*Organic calcareous soils (Calcic Murshic Histosols)—Soc*															
	*Ružomberok-Podsucha 1*	35.54		25.38	2.04	0.59	12.53	7.03		6.80	676.00	172.7	70.73	118.6	54,442.5	2107.5
	*Ružomberok-Podsucha 2*	37.93		27.41	1.94	0.42	14.66	7.07		6.88	669.00	154	111.23	126.23	126,150	1944
	*Ružomberok-Podsucha 3*	34.38		21.74	1.11	0.19	21.06	7.11		6.91	673.50	148.5	111.64	142.1	131,800	2027
	$\bar{x}$	35.95b		24.84b	1.69 cd	0.40a	16.08a	7.07b		6.86d	672.83a	158.40a	97.87a	128.98ab	104,130.83b	2026.17c

*Carex pulicaris* was also found on moist mineral soils (Umbric Gleysols) with a mean organic matter content of 10 to 16% and a C/N ratio ranging from 13 to 14. Despite their similar systematic affiliation, the surface soil layers (rhizospheres) differed in their chemical composition between Estonia, Denmark and Poland (Table 2). The soils in Estonia were slightly acidic, with a low content of assimilable phosphorus and potassium but with an abundance of Mg. On the other hand, soils from Denmark and Poland were acidic and poor in assimilable K and Mg; however, the soils from Estonia and Denmark demonstrated significantly lower assimilable P and considerably lower exchangeable Na content than those from Poland. Moreover, all three groups of soils were characterized by a low salt concentration and a similar content of exchangeable Ca. All analyzed soils contained small amounts of bioavailable and total forms of heavy metals (Cd, Co, Cu, Pb, Ni, Zn, Mn and Fe; Tables 3 and 4).

**Table 3:** Content of available heavy metals in soils.

The localizations of *Carex pulicaris*		Available heavy metals (soluble in 0.5 mol dm^−3^ HCl)							
		Cd	Co	Cu	Ni	Pb	Mn	Fe	Zn
		mg kg^−1^							
Estonia	*Organic soils (Murshic Histosols)*								
	*Laukna 1*	0.46	0.5	2.8	2.37	8.53	20.56	1901.33	7.32
	*Mullavere*	0.13	0.32	0.63	0.36	3.37	30.72	1299.9	6.61
	*Pȕhatu*	0.29	0.38	0.75	0.36	9.63	21.34	3376	10.94
	$\bar{x}$	0.29ac	0.40ab	1.39ab	1.03a	7.18a	24.21ab	2192.41ab	8.29a
	*Mineral soils (Umbric Gleysols)—Em*								
	*Laukna 2*	0.06	0.53	1.57	0.45	7.61	10.1	1163	5.27
	*Mahtra*	0.14	0.68	2.7	1.31	5.79	20.87	1438	9.15
	*Ȕdruma*	0.17	1.39	1.55	0.71	8.59	41.07	2337.67	15.02
	$\bar{x}$	0.12c	0.87ab	1.94ab	0.82a	7.33a	24.01ab	1646.22ab	9.81a
Denmark (Bornholm)	*Mineral soils (Umbric Gleysols)—Dm*								
	*Sandviq*	0.37	0.51	1.05	0.16	8.93	77.74	893	2.89
	*Ravnekaer Lake*	0.79	1.1	6.67	1.6	18.38	159.4	1158	14.37
	*Bastemosse*	0.41	1.05	7.55	1.54	14.2	44.06	1000	7.01
	$\bar{x}$	0.52ab	0.89ab	5.09b	1.10a	13.84a	93.73b	1017.00ab	8.09a
Poland	*Organic soils (Murshic Histosols)—Po*								
	*Domysłów*	0.16	0.01	0.88	0.54	6.82	42.45	2617	23.47
	*Rozwarowskie Marsh*	0.47	0.21	2.56	0.13	35.6	14.46	1309	36.57
	*Beka*	0.27	0.01	1.27	0.79	4.87	3.57	178.9	5.28
	$\bar{x}$	0.30abc	0.08a	1.57ab	0.49a	15.76a	20.16a	1368.30ab	21.77ab
	*Mineral soils (Umbric Gleysols)—Pm*								
	*Nosalin*	0.21	0.02	2	1.36	0.56	13.49	662.2	23.93
	*Piaśnickie Łąki*	0.37	0.1	2.23	1.31	6.57	6.33	996.4	11.18
	*Polana Białego Potoku*	0.52	0.97	2.76	1.66	14.29	27.75	1565	28.26
	$\bar{x}$	0.37abc	0.36ab	2.33ab	1.44a	7.14a	15.86a	1074.53ab	21.12ab
Slovakia	*Organic soils (Murshic Histosols)—So*								
	*NP Poloniny NPR 1*	0.7	1.08	11.03	9.74	24.31	67.48	2664.55	16.66
	*NP Poloniny NPR 2*	0.67	1.04	10.59	9.12	23.07	61.7	2387.3	15.62
	*NP Poloniny NPR 3*	0.68	1.03	10.39	9.03	22.73	62.72	2285.05	16.72
	$\bar{x}$	0.68b	1.05b	10.67c	9.30b	23.37a	63.97ab	2445.63b	16.33a
	*Organic calcareous soils (Calcic Murshic Histosols)—Soc*								
	*Ružomberok-Podsucha 1*	0.57	0.4	0.4	0.71	0.23	234.76	228.31	41.6
	*Ružomberok-Podsucha 2*	0.59	0.49	0.8	0.84	8.29	254.18	447.19	39.24
	*Ružomberok-Podsucha 3*	0.58	0.34	0.36	0.73	4.27	253.74	213.99	40.82
	$\bar{x}$	0.58ab	0.41ab	0.52a	0.76a	4.26a	247.56c	296.50a	40.55b

**Table 4:** Total content of macroelements and heavy metals in the soil.

The localizations of *Carex pulicaris*		Total macro and heavy metals											
		P	Na	K	Ca	Mg	Cd	Co	Cu	Ni	Pb	Mn	Zn
		mg kg^−1^											
Estonia	*Organic soils (Murshic Histosols)—Eo*												
	*Laukna 1*	808.5	1176.4	1391.63	40,536.67	1691.67	1.48	5.41	5.24	2.91	11.62	54.96	23.09
	*Mullavere*	960.67	803.83	2062.67	39,478.33	4238.33	0.49	14.76	9.34	4.31	15.54	126.44	34.45
	*Pȕhatu*	1598.67	661.73	1849.83	26,120	1646.67	0.53	5.17	5.97	0.69	12.08	42.4	15.48
	$\bar{x}$	1122.61abc	880.65a	1768.04a	35,378.33a	2525.56a	0.83a	8.45a	6.85ab	2.64a	13.08a	74.60ab	24.34a
	*Mineral soils (Umbric Gleysols)—Em*												
	*Laukna 2*	1287	674.6	1608.75	5868	1603.50	0.16	10.04	2.69	2.84	10.16	98.31	18.5
	*Mahtra*	850.67	1590.97	3039.17	9032.5	1089.75	0.54	6.68	7.54	1.66	10.21	59.11	21.46
	*Ȕdruma*	1094.5	1032.6	2270.13	11,386.67	1445.67	0.26	9.86	4.64	1.68	22.53	165.2	47.36
	$\bar{x}$	1077.39abc	1099.39a	2306.02a	8762.39a	1379.64a	0.32a	8.86a	4.96ab	2.06a	14.30a	107.54ab	29.11a
Denmark (Bornholm)	*Mineral soils (Umbric Gleysols)—Dm*												
	*Sandviq*	792	505.8	2133.5	644.9	212.5	0.9	4.23	7.44	0.28	21.6	163	27.99
	*Ravnekaer Lake*	792	2855	1372.5	3748	3448	0.9	4.72	11.06	5.06	37	190.6	75.1
	*Bastemosse*	660	2638	6660	8525	789.5	0.81	3.67	9.22	2.39	38.36	164.5	15.74
	$\bar{x}$	748.00ab	1999.60a	3388.67a	4305.97a	1483.33a	0.87a	4.21a	9.24a	2.58a	32.32ab	172.70b	39.61abc
Poland	*Organic soils (Murshic Histosols)—Po*												
	*Domysłów*	924	2331	321	22,500	749.5	0.55	2.64	7.56	3.59	23.71	98.9	70.34
	*Rozwarowskie Marsh*	1716	3604	5005	8404	1002.5	0.55	4.83	3.08	2.27	46.48	121.6	39.19
	*Beka*	1716	1024.8	2895	18,276.7	3378.6	0.4	8.4	5.49	1.92	41.6	154.5	50.21
	$\bar{x}$	1452.00bc	2319.93a	2740.33a	16,393.57a	1710.20a	0.50a	5.29a	5.38ab	2.59a	37.26b	125.00ab	53.25abc
	*Mineral soils (Umbric Gleysols)—Pm*												
	*Nosalin*	2024	2012	1872.5	8990	1410	0.45	6.06	3.35	3.79	35.74	60.61	20.81
	*Piaśnickie Łąki*	1342	2200	1401	10,970	1379	0. 75	6.79	2.28	2.64	21.5	63.56	30.08
	*Polana Białego Potoku*	1430	1906	2879.5	5571	768	1.44	10.34	3.96	4.24	30.62	31.44	50.72
	$\bar{x}$	1598.67c	2039.33a	2051.00a	8510.33a	1185.67a	0.95a	7.73a	3.20b	3.56a	29.29ab	51.87a	33.87ab
Slovakia	*Organic soils (Murshic Histosols)—So*												
	*NP Poloniny NPR 1*	369.05	434.26	715.35	15,250	2673.15	0.98	6.94	25.27	22.48	36.65	99.71	80.63
	*NP Poloniny NPR 2*	378.4	458.75	529.45	14,710	2530.5	0.88	7.48	24.12	21.23	26.26	91.93	70.62
	*NP Poloniny NPR 3*	371.8	620.29	576.55	14,695	2477	0.95	8.44	25.36	23.52	30.41	94.16	81.35
	$\bar{x}$	373.08a	504.43a	607.12a	14,885.00a	2560.22a	0.94a	7.62a	24.92c	22.41a	31.11ab	95.27ab	77.53bc
	*Organic calcareous soils (Calcic Murshic Histosols)—Soc*												
	*Ružomberok-Podsucha 1*	343.75	225.69	1330.25	135,750	3190.53	1.42	10.68	9.26	4.09	36.79	354.75	85.55
	*Ružomberok-Podsucha 2*	333.85	229.74	1040.55	362,418	2881.83	1.07	6.06	7.88	3.43	31.61	261.35	78.39
	*Ružomberok-Podsucha 3*	312.95	496.23	1099.75	173,071.5	3062.01	1.27	7.67	9.12	4.33	37.48	355.9	86.46
	$\bar{x}$	330.18a	317.22a	1156.85a	223,746.50b	3044.79a	1.25a	8.14a	8.75a	3.95b	35.29b	324.00c	83.47c

**Figure 6:**
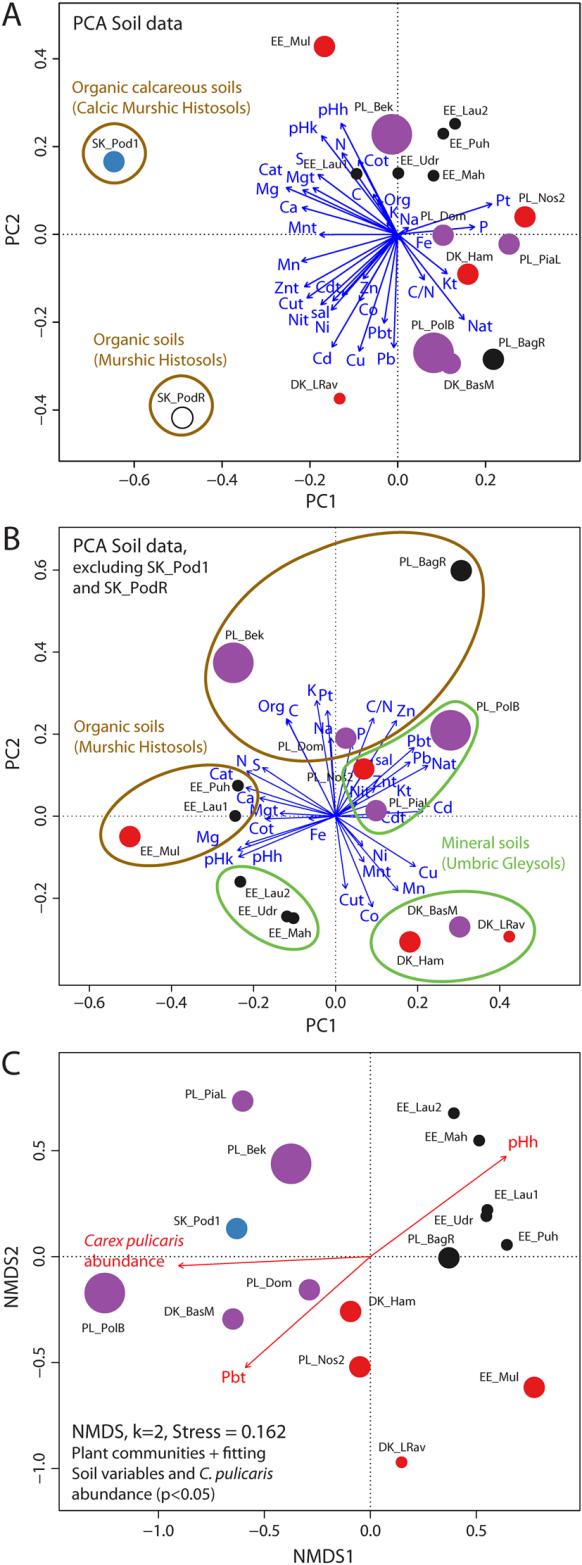
Ordination biplot of principal component analysis (PCA) of soil dataset. (**A**) including Slovak sites SK_Pod1 and SK_PodR and (**B**) excluding them (lower panel). Grouping circles according to classically recognised soil types; (**C**) non-metric multidimensional scaling (NMDS) of species composition data showing grouping of localities, with maximal variance explained along NMDS1 and NMDS2. Soil variables significant at p < 0.05 fitted to ordination biplot. Locality abbreviations follow Table 1. Colouring corresponds to clustering pattern, symbol size reflects *Carex pulicaris* abundance. *Carex pulicaris* abundance is not related to PCA grouping patterns (p > 0.05) but significant to plant community grouping (p < 0.05). Colouring of symbols: black—Estonian group, blue—Slovakian group, orange—Radecz group, purple—Ambiguous group, red—Polish group, white—locality in Slovakia with the occurence of *C. pulicaris*, but lacks species composition data. Note: Averages from three subsamples were inputs for localities SK_Pod1 and SK_PodR.

The PCA analysis of the soil dataset (17 sites × 34 soil variables) found that the site groupings reflect generally geographic locations and soil types; in addition, this patterning appears to be irrespective of plant community type, and of the abundance of *C. pulicaris* (Fig. 6A,B). In contrast, the results of the NMDS analysis of plant communities with regard to soil subset (16 sites × 194 species, plant community data are missing for single locality from Slovakia—SK_PodR) are roughly in line with the relevé grouping in the whole dataset (43 sites × 310 species). NMDS analysis separated the Estonian and Polish vegetation groups, on the right of the plot, from the Ambiguous group and one Slovakian relevé, on the left of the plot (Fig. 6C). The only soil variables significantly fitted to this NMDS grouping were pH and total Pb. Most importantly, *C. pulicaris* abundance fits significantly to the NMDS groupings (p < 0.01) (Fig. 6C).

## Discussion

The Estonian, Slovak and Radecz groups, which were most closely related to a particular region within the range of *C. pulicaris*, were characterized by a homogeneous type of vegetation. The Estonian group was represented by communities of the Molinion caeruleae alliance, sometimes with a significant share of *Carex davallianae*, and the other two by communities of the Caricetalia davallianae order. In Slovakia, *C. pulicaris* is associated with non-forest communities of minerotrophic fens, particularly with the Sphagno warnstorfiani-Tomenthypnion alliance^29^. In turn, the Polish and Ambiguous groups showed greater diversity: their composition included communities from both of the above-mentioned syntaxonomic units, with the Polish group including some from the Caricetalia nigrae order, with some tendency towards the Nardo-Callunetea class.

Throughout its entire range, *C. pulicaris* is commonly derived from the communities of the Molinion alliance, and this can be seen in Slovenia, the Netherlands and Sweden^7,44,45^. In France, it was recorded in acidic fen grasslands (Caricetalia nigrae communities)^46^, and in Great Britain, in communities such as calcicolous grassland community *Sesleria albicans*—*Galium sterneri* in the *Carex pulicaris*—*Carex panicea* subcommunity, mire community *Carex dioica*—*Pinguicula vulgaris* and *Molinia caerulea*—*Cirsium dissectum* fen-meadow (according to the UK NVC^47^). The last of these communities is the British equivalent of *Cirsio-Molinietum* Sissingh & de Vries 1942, described from the Netherlands, Belgium, Germany and Ireland^48^.

The groups of plant communities separated as a result of the analyses were generally characterized by considerable plant species diversity, with the only exception being the Estonian group (Fig. 3); the lower diversity indices in this group was undoubtedly influenced by the high share of *Molinia caerulea* in the studied phytocoenoses. This is a highly competitive grass, and its presence in large clumps not only limited the number of safe places for the germination of other plant species diaspores, but also hindered their development. This was especially true for annual plants, and to a lesser extent, the perennials, which show vegetative reproduction. Moreover, most of the plant patches were partially shaded by the crowns of trees growing in the immediate vicinity. Under these conditions, *C. pulicaris* often grew in the *Molinia* clumps. We observed this phenomenon not only in the locations in Estonia, but also in the Rozwarowskie Marshes in Poland (PL_BagR).

Regardless of the research area, *C. pulicaris* was often accompanied by species associated with grassland communities, particularly meso- and eutrophic hay meadows and riverside herbaceous plant communities; these are permanently, or at least periodically, moist (order Molinietalia caeruleae). The significant share of these species in some of the examined patches of *C. pulicaris* communities from the Caricetalia davallianae order (e.g. in Slovakia) may indicate the presence of fluctuations in the water regime and/or a slightly increased availability of nutrients for plants. Such coexistence of species may take place over a longer period of time, and in the case of a permanent change in habitat conditions, it may lead to another stage of succession towards meadow communities from the Molinion caeruleae alliance^29^. Hence, it is sometimes difficult to unequivocally define the syntaxonomic affiliation of plant communities from *C. pulicaris.*

The share of *C. pulicaris* in the analyzed groups was diversified, and it was neither a dominant nor a codominant species in any of the phytocoenoses. In contrast to the eastern end of its range, *C. pulicaris* can be found abundantly in the remainder of its distribution area; it has been reported as the dominant or codominant species in some phytocoenoses, for instance in Great Britain and Ireland^47,49,50^. As *C. pulicaris* is a poorly-germinating^51^, light-requiring species that is weak in competition, its abundance is favored by extensive land use, which limits the development of tall, expansive perennials. Our findings indicate that the mowing of meadows in areas where *C. pulicaris* occurs (Domysłów and Rozwarowskie Marshes) favors its survival, even under conditions of high competition from *Molinia caerulea*, and in the vicinity of *Phragmites australis* plantations. It is classified as a species dependent on mowing in the area of the fenoscandian limestone forest meadows^52^, and was only found in mowed areas in the costal grasslands in the Stockholm archipelago^45^. Also in the Netherlands, this type of land use is necessary to maintain the structure and species composition of meadows belonging to the Molinion caeruleae alliance, where *C. pulicaris* is present^7^. This prevents the succession towards scrub and forest, especially when the habitat is fragmented.

Our findings also indicate that a higher abundance of *C. pulicaris* is correlated with greater precipitation, albeit to a small extent (Fig. 5). This relationship is demonstrated by the fact that the species was most abundant at the Polana Biały Potok (Tatry Mts) site (PL_PolB), which had the highest mean annual precipitation (915 mm) of the examined sites. *Carex pulicaris* is a sub-Atlantic ranging type^53^, indicating that it favors areas with a significant amount of rainfall. It is a common species in Great Britain, being sometimes codominant in plant communities, and is often observed in densely-tufted patches with *Sesleria albicans*^47^; in this country, it has been found in areas with an annual rainfall of 1239 mm^54^.

An important factor limiting the range of *C. pulicaris*, as well as other sub-Atlantic species, may be the occurrence of winter frost^55^. Comparing the isotherms of the mean month temperatures of the coldest month (°C) calculated for sea level^55^ with the species distribution data^13,53^, a clear relationship can be seen between the limit of its range and the -4 °C isotherm. The results of local studies from mountain areas in southern Germany^14^ highlight the significant role played by both temperature and precipitation on the occurrence of *C. pulicaris*. In contrast, while our present findings do not show any clear relationship between species abundance and air temperature, the CCA analysis (Fig. 5B) suggests that rainfall is of greater importance.

Our results suggest that in the eastern edge of the *C. pulicaris* distribution range, its abundance may be latitude dependent, with a lower abundance observed in plant communities in more northerly locations. In addition, it grew sparingly in the north-eastern part of our research area, including almost all the Estonian sites, but had a fairly significant share in the community patches in the south-east, including most Slovak sites. However, no such relationship was observed in the north-western extremities of its range, where in some plant communities, it is very common and is the dominant species^49^; it should be noted though that, being a sub-Atlantic species, the conditions for development in these areas are very favorable.

*Carex pulicaris* is found on various types of bogs, including fen, transitional and lagg of raised bogs, as well as wet heathlands, *Nardus* grasslands and wet meadows^56–58^. In addition to organic soils, it also often inhabits humus and moist mineral soils^44,56^, while it is less common in other habitats, such as clay or sandy peat soils^59^. In the sites in the present study, *C. pulicaris* grew in humid but diversified habitats: from bogs, where the *Murshic Histosols* soils developed, to humus mineral *Umbric Gleysols* soils^38^, regardless of whether it was at the edge or the center of the distribution range. *Carex pulicaris* occurs on a variety of soil types with organic matter content ranging from 1.6 to nearly 100%^49^.

Certain chemical and physical properties of soils have a strong influence on the occurrence of plants^60^. Our research showed that the surface layers (rhizospheres) of the soils in the tested areas demonstrated great variation in their physical and chemical compositions. *Murshic Histosols* were acidic to slightly acidic, while *Umbric Gleysols* were acidic to neutral^38^.

The areas across Europe where *C. pulicaris* occurs demonstrate similar variations in soil pH^44,49,57,58,61^. However, *C. pulicaris* generally preferred slightly acidic soils, and did not favour strongly acidic and alkaline soils. Our study showed that soil pH was significantly correlated with the grouping of plant communities with *C. pulicaris* participation. Soil acidity affects such features as the trophic nature of the habitat, the quantity and quality of humus, and the abundance of assimilable macro- and microelements. The wide range of soil pH values tolerated by *C. pulicaris* may indicate a well-developed adaptability to various environmental conditions; however, this contrasts with previous observations, i.e. that the species occurs in bogs of a narrow ecological amplitude^29^.

Nitrogen, K and P levels are particularly important for plant community stability. In wet habitats, an increase of P, K and N levels in soil can lead to a decline in the diversity and abundance of plant species^44,62–64^. The results of our analyses showed that both organic and mineral soils were generally low in available P and K, which may support the growth of *C. pulicaris*. These habitats have previously been found to have low nitrogen content^44,65^, and data from areas within the *C. pulicaris* range suggest that its communities require low N, P and K concentrations^44,61,62,65–69^; however, our present results do not indicate any significant relationship between the abundance of *C. pulicaris* itself and the P, N and K content of the soil. The plant communities of *C. pulicaris* are sensitive to excess N-NH_4_ levels and N-NO_3_ deficiency; this relationship is related to long-term stagnation of water on the soil surface, resulting in the development of anaerobic conditions^70^.

Our own studies indicate that *C. pulicaris* abundance is not significantly affected by soil Ca, Mg and salt content. The species was recorded in soils with various Mg, Ca and salinity levels^44,65^, and its associated plant communities have been observed in habitats relatively rich in nutrients^29,49,65,67^. It has also been found to grow in soils containing low levels of heavy metals^39,40^.

*Carex pulicaris* appears quite resistant to anthropogenic influences known to increase habitat trophy, as indicated by our finding that macro- and micronutrient content appears to have no clear effect on its abundance in plant communities. On the other hand, distinct changes in soil moisture and the cessation of pratotechnical practices may limit its abundance^44,70^.

*Carex pulicaris* responds well to surface floods in the autumn-spring period as well as to strong falls in groundwater level in summer^71^. Short periodic floods cause a reduction in the mineralization of organic matter; they also drive the release of nutrients, which are beneficial for the habitat, and stimulate the formation of Fe, Ca and P complexes, which are normally inaccessible to plants^72^. However, if maintained for too long, excessive soil moisture levels result in changes in the oxidoreductive and chemical properties of the soil that are unfavorable for the growth of *C. pulicaris*^70^. In contrast, a rise in the level of groundwater may favor paludification processes, which in turn will lead to the disappearance of *C. pulicaris* as a result of the reconstruction of plant communities with more competitive members. In addition, excessive dehydration and oxygenation of the habitat causes rapid mineralization of organic matter and the release of large quantities of mineral nutrients; this is also unfavorable for *C. pulicaris*, and favors the entry of expansive perennials, trees and shrubs.

Finally, most of the Estonian sites were characterized by large shares of trees in the vegetation patches; this can increase shading and thus may be the reason for the low abundance of *C. pulicaris*. The specific humidity requirements described above explain the sensitivity of this species to changes in humidity in the habitat.

## Conclusions

Within the eastern end of its range, *C. pulicaris* is present in five natural phytocoenological groups: four closely correspond to geographic regions, viz*.* Estonia, Poland, Slovakia and Radecz (Poland), and a fifth Ambiguous group scattered throughout the study region. In general, the groups show a significant diversity of species composition. The Estonian group was represented by communities from the Molinion caeruleae alliance, the Slovak and Radecz groups by communities from the Caricetalia davallianae order, while the other two showed greater diversity with regard to their syntaxonomic units. However, *C. pulicaris* was not dominant or codominant in any of the groups with regard to the studied phytocoenoses.

Our findings indicate that the abundance of *C. pulicaris* is positively correlated with the composition of its geographically-diversified plant communities and atmospheric precipitation. In the eastern part of its range, *C. pulicaris* grows on organic (Murshic Histosols) and mineral (Umbric Gleysols) soils associated with high groundwater conditions. These soils are characterized by an acidic to neutral reaction, narrow C:N ratio, low salinity, mostly low levels of available P and K and micronutrients, and varying levels of available Mg, Ca and Na. *Carex pulicaris* appeared to grow most abundantly in plant communities which prefer slightly acid soils.

## Data Availability

All data matrices that support the findings of this study are available from the corresponding author upon request.
